# A Qualitative Study of the Systems-Based Practice Competency in United States Graduate Medical Education

**DOI:** 10.7759/cureus.109540

**Published:** 2026-05-24

**Authors:** Ami L DeWaters, Erin Miller, Nate Long, Paul Haidet, Jed Gonzalo

**Affiliations:** 1 Internal Medicine, Penn State College of Medicine, Hershey, USA; 2 Biostatistics, RTI Health Solutions, Hershey, USA; 3 Medicine, MaineHealth, Portland, USA; 4 Medicine, Penn State College of Medicine, Hershey, USA; 5 Internal Medicine, Virginia Tech Carilion School of Medicine, Roanoke, USA

**Keywords:** co-production, core competency, graduate medical education (gme), learning health systems, qualitative studies

## Abstract

Background

Systems-based practice (SBP) is a core competency in U.S. graduate medical education (GME), but it is not well implemented; we examined current factors that need to be addressed to advance SBP.

Methods

Between March and July 2021, 24 physicians, nurses, educators, and leaders in the field of SBP were interviewed using a semi-structured guide. Individuals were selected based on their influence on the origin or development of SBP. The authors iteratively collected and analyzed data using real-time analytic memos, regular adjudication sessions, and thematic analysis. Themes were agreed upon by the research team.

Results

Four themes were identified: (1) co-production is a central concept in the implementation of SBP, (2) co-production needs to occur between multiple stakeholders across a multitude of settings, (3) health organizations need to transform into learning health organizations to advance SBP, and (4) macrosystem-level fragmentation and organizational culture are inhibiting SBP in healthcare delivery.

Conclusions

The ability of GME programs to successfully implement SBP is closely tied to their ability to collaborate with their local health systems and have those health systems actively support SBP principles. Co-production and transformation into learning health systems are two key strategies for achieving optimized SBP.

## Introduction

In 1997, the Accreditation Council for Graduate Medical Education (ACGME) began the Outcomes Project, which facilitated the integration of the assessment of educational outcomes into United States (US) graduate medical education (GME) [1]. During subsequent years, the ACGME established six core competencies in GME, one of which was systems-based practice (SBP) [2]. SBP includes the domains of advocacy, teaming, coordination of care, patient safety, quality improvement, and value-based care [3]. The inclusion of SBP was viewed as the ACGME’s acknowledgement of “the critical need for direct attention to navigating the system itself as a key component for successful and effective patient care” [4].

SBP has been described by several individuals who established it as one of the most difficult competencies to operationalize, and it is challenged by the perception that it is an abstract and obscure concept [1,4,5]. In addition, there have been recent calls for SBP to include more attention to domains such as healthcare inequities and social determinants of health, increasing the complexity of the discussion about the scope of SBP [6,7]. Given the challenge of understanding SBP, it is not surprising that GME programs struggle to successfully implement SBP in training [4,8-12]. In addition, there is both variability and inconsistency in how SBP sub-competencies are taught among GME programs [4]. Prior literature has highlighted the cultural, institutional, and resource barriers that preclude programs’ ability to comply with ACGME SBP mandates, thereby further hindering SBP implementation [4].

While the challenges of SBP implementation are known, there is a lack of literature exploring the ways in which SBP could be operationalized more effectively, particularly from the perspective of the individuals who originally established the competency and have been actively working to evolve it over the last 20 years. The first aim of this study was to explore these experts’ perspectives on what factors could advance the SBP competency in today’s GME programs. The second aim was to explore the barriers that hinder SBP advancement.

## Materials and methods

Study approach and setting

We approached this research study using a social constructivist worldview. Therefore, we performed in-depth, individual interviews with our participants. Our research team sought to identify our own biases and articulated our common beliefs that SBP is critical for the evolution of medical education and that SBP has not been well implemented in GME programs over the last 20 years. We accounted for these biases during the development of the interview guide to avoid leading participants. These biases were primarily addressed using collaborative reflection in research team meetings that were explicitly focused on ensuring reflexivity. Additionally, we performed negative case searches and crosschecks of emergent themes in our data analysis in an effort to manage our beliefs and avoid undue influence on our conclusions. We followed qualitative research reporting standards [13-18]. Of note, this study also resulted in previously published findings [19]. The study was designated exempt by the Institutional Review Board (Penn State College of Medicine Institutional Review Board protocol STUDY00015764).

Participant sampling

We identified participants for potential inclusion in the study who were influential in either the establishment or evolution of SBP, or both. Table 1 outlines the inclusion and exclusion criteria. We reviewed online websites, publications, and textbooks to develop a potential list of participants, and the preliminary list was then revised by an SBP leader (Dr. Eric Holmboe). We deliberately chose participants from diverse specialties and roles to achieve a comprehensive assessment of SBP implementation. We did not include current “frontline” program leaders and learners, as the aim of this study was to leverage historical perspectives to gain insight into the improvement of SBP. Recruitment was performed via email, which described the study and invited participants for an interview.

**Table 1:** Inclusion and exclusion criteria for participants in a study on the development of the systems-based practice competency. SBP: Systems-based practice.

Inclusion criteria	Exclusion criteria
Influential in the establishment of SBP as a core competency	“Frontline” program leaders, such as program directors and associate program directors
Influential in early attempts to define SBP	Trainees, such as medical students or residents
Influential in early attempts to teach SBP	Medical education leaders without expertise in SBP
Influential in early attempts to assess SBP	
Influential in describing current challenges with SBP	
Influential in describing interprofessional views of SBP	
Influential in establishing international views of SBP	

Data collection methods, instruments, and technology

After consenting participants, we performed audio- and video-recorded Zoom™ interviews, ranging in length from 25 to 85 minutes. Interviews were performed between March and July 2021. The research team developed an interview guide, which used open-ended questions to explicate participants’ perspectives about SBP (Appendix 1). Categories of questions included the history and origin of SBP, SBP teaching and learning, system citizenship or professional identity of physicians, the relationship of SBP to the clinical learning environment, and the future of SBP. At the start of each interview, we obtained informed consent from each participant. During each interview, one to three co-investigators observed and intermittently asked further clarifying questions. Transcriptions of the interviews were provided through Penn State University’s professional Zoom™ service. These were checked for accuracy by a co-investigator (E.M.). No incentives were offered to the participants.

Data analysis 

Data were analyzed using thematic analysis [13-16,18]. One investigator (J.D.G.) recorded analytical memos in real time while watching interviews, which were then used during team discussions to generate a preliminary codebook. The research team then selected nine transcripts for each member of the team to review individually. All team members then engaged in hours of group discussion, spaced over two months, to identify initial themes and refine the preliminary codebook. Following this, each investigator reviewed an additional set of three transcripts and discussed these in additional meetings. When this process did not identify any further codes or categories, two investigators (A.D. and N.L.) used thematic analysis to independently code each transcript with the developed codebook. A third investigator (E.M.) reviewed all data and preliminary categories as provided by the independent coders. The team met regularly to discuss findings, reconcile disagreements, and agree upon final themes and quotations. The discussions in these meetings also included collaborative reflections to help us engage in reflexivity and challenge our own biases. Quotes were corrected for grammatical errors, and, at times, syntax was changed to improve readability. The data were managed with support from NVivo™.

## Results

We interviewed 24 individuals who collectively represented all categories of SBP creation and development. Fourteen participants were men (58%), and 10 were women (42%). Participants represented all phases of the origin and evolution of SBP, with at least two individuals included from each of the five possible categories. Four key themes related to the improved implementation of SBP emerged: co-production is a central concept in the implementation of SBP; SBP implementation requires co-production between multiple stakeholders across a multitude of settings; health organizations need to transform into learning health systems to advance SBP; and macrosystem-level fragmentation and organizational culture are the primary inhibiting factors for SBP implementation.

Theme 1: Co-production is a central concept in the implementation of SBP

The participants nearly universally pointed toward the need for co-production to enhance SBP implementation in GME. Co-production has been defined as “the contribution of service users to the provision of services” [20]. Patients assisting health systems in building self-management programs for individuals with chronic illnesses, such as diabetes, would be an example of co-production [20]. As two experts stated:

“You have to understand that a service is always co-produced in some way, and health care is a service. It’s made by both parties, two parties come together, they are interdependent. And together they create a service and that co-creation process or that co-production process is when healthcare works well.” 

“We were talking about co-production of care and it’s a big thing people in healthcare work with patients so that patients can co-produce their own care…I think it’s critical because it affects [patients’] outcomes, affects [patients’] satisfaction, you could save a life if you did that.” 

Another participant commented on the need for co-production and the inclusion of resident physicians in the process:

“My ideal state is that all this is done through the lens of co-production. Residents are partners. They’re learning with faculty but they’re also contributing. Co-production becomes just a normal mindset moving forward [with SBP].” 

This participant echoed the above statements by saying:

“Looking at education from a co-production lens where teacher and learner or healthcare provider and patient in the co-production health care model will help us to produce the learners that we want for the 21st century.” 

Theme 2: SBP implementation requires co-production between multiple stakeholders across a multitude of settings

**Figure 1:**
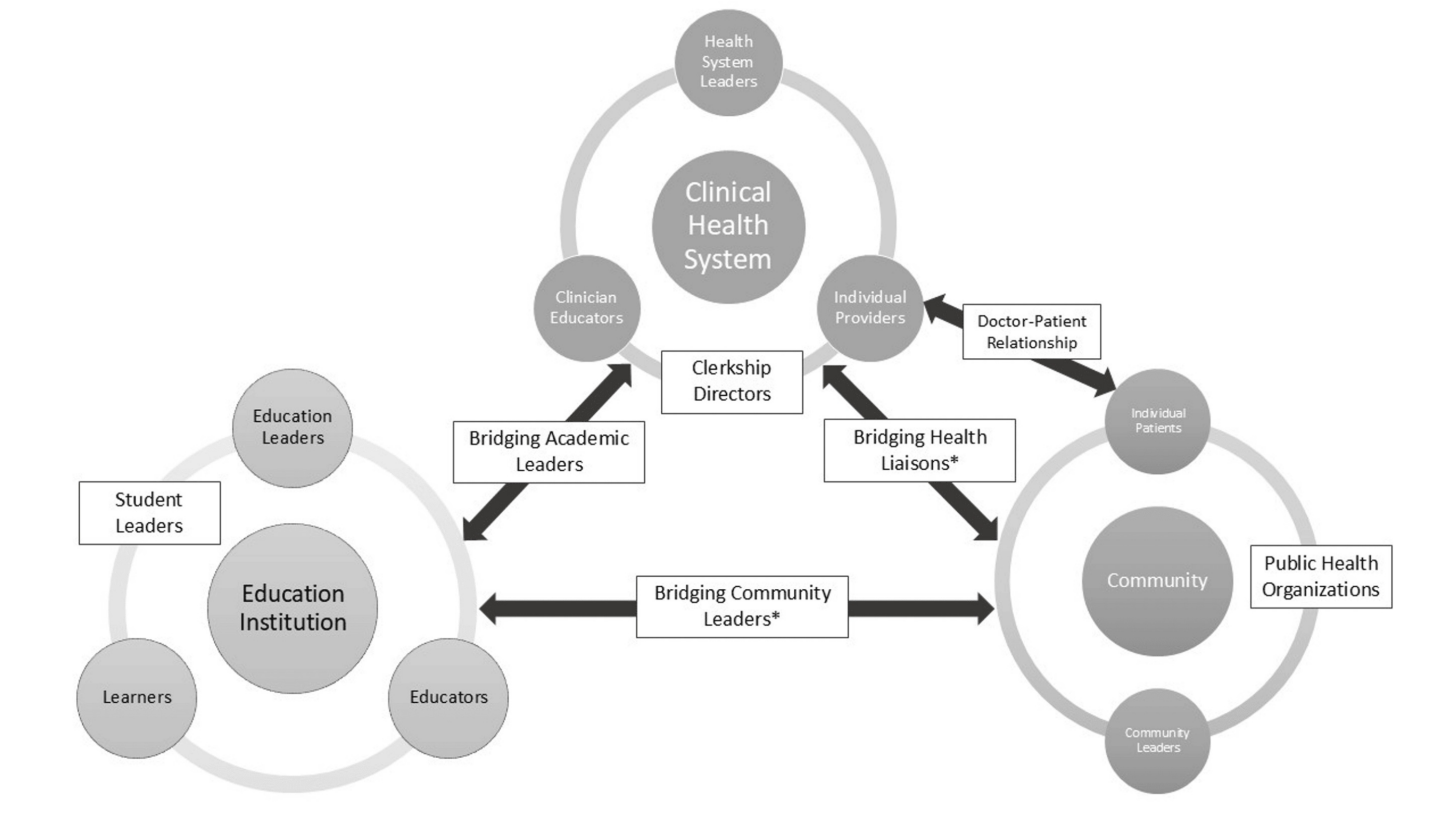
Co-production partnerships and roles to enhance co-production in an academic health system. *This is a new role that does not traditionally exist in current academic health systems. Note: This image was created in PowerPoint by the authors of the study. It was not generated using AI.

In addition to mentioning the importance of the general concept of co-production to advance SBP, participants referred to a range of possible co-production partners across a variety of settings who needed to engage in the co-production process. Figure 1 summarizes the different partnerships and settings suggested for co-production. The different partnerships highlighted a variety of “users” of SBP. One of the most frequently mentioned partnerships was that of learners partnered with organizational leaders from the health system, where the user was defined as a learner or resident. One interviewee explained:

“We talked a lot about ‘How do we bridge the gap between the learning environment and training programs? How can you create structural changes and how can you create certain process changes? Structural changes might be to have learners’ representatives on organizational committees, where there’s the opportunity to influence decisions around how the organization implements certain changes. And then, the degree to which people are co-creating the learning experiences.” 

Another interviewee described the potential role of health system leaders in informing the design of SBP learning experiences for students and residents:

“A question you could always ask is ‘If you’ve got a curriculum on SBP, to what extent do the organizational leaders have a role in helping to design those experiences to happen? To what extent are they involved in delivering any of that content? To what extent is the organization providing opportunities for the learners to explore these concepts within the clinical learning environment? I think there are ways that if you ask some of these questions, you can start to get a sense as to whether or not the clinical environment, the leadership, and the providers within that setting are really meaningfully involved in shaping the learning [of SBP], or more passively involved.” 

Another frequently mentioned partnership was that between health systems and local community governments. The participants described a strong need to increase attention to population health management to advance SBP. One interviewee described an example program that could advance SBP:

“They do county councils that are responsible for the population health… so they integrate the university and administration and the government business with the medical field and health professionals around improvement science and SBP and then their outcomes are outstanding.” 

Finally, the idea of “bridging leaders” was described by several participants as a necessary role to foster co-production for SBP. One interviewee mentioned:

“Solid leadership, both within educational environments and within healthcare systems, and alignment across leadership groups support [SBP]… you know there’s a home for basic sciences and a home for clinical sciences but there isn’t really a home for an organizational leader that advocates for that SBP domain.” 

This was supported by another participant who stated, “Every system doesn’t always have the right way to evaluate these things [SBP], so I have talked about bridging leaders to facilitate this.”

Theme 3: Health organizations need to transform into learning health systems to advance SBP

Frequently, participants connected their comments on co-production to a larger vision for health organizations to become learning health systems. A learning health system is defined as having science, informatics, incentives, and culture aligned for continuous improvement and innovation, with best practices seamlessly embedded in the delivery process [21]. One interviewee illustrated the necessity of creating learning health systems and the relation to co-production by describing a program that had reduced relapse rates for a chronic disease by over 50% and stated, “Isn’t that amazing? So, what we do to achieve that is we create a framework and we call it a learning health system for co-producing better health outcomes.”

Several participants spoke about the need for facilitated learning networks that would exist nationally or even internationally to evolve SBP, rather than the development of local learning health systems alone. One participant stated, “National collaborative learning networks are going to be the future of healthcare. If I’m a patient and [say], ‘Tell me the quality of the care you deliver as a care team,’ [national collaborative learning networks] can tell you how [they are] improving care. Building the system for this is absolutely critical [for SBP].” Another participant added:

“It’s the international co-production learning health system that would be a great example, so being in an environment where that microsystem is great and that microsystem is taking part in a broader co-production learning health system…that would be the best care.” 

Theme 4: Macrosystem-level fragmentation and organizational culture are the primary inhibiting factors for SBP implementation

The final theme that emerged was a belief that the macrosystem of health care, i.e., healthcare systems at the national level, is so fractured that advancing SBP becomes difficult. One interviewee declared in response to advancing SBP, “You’re fooling yourself because we don’t really have a system [of healthcare].” Another stated that to advance SBP:

“We’d need to establish a healthcare system that works with other systems, like social care and housing, all the things that affect health. One of the things we have noticed in many countries is the lack of dialogue between the ministry of higher education and the [healthcare] system delivery. I’ve seen in many countries [the groups] not necessarily talking to each other or making compromises. That comes to a crisis because we could have produced a first-class health professional but [they are] not regarded as such by the potential utilizers of the product [of healthcare]. That is an important mismatch.” 

Participants discussed the role of organizational culture and subsequent leadership support in hampering or facilitating SBP teaching and learning. Specifically, participants discussed how SBP can be facilitated when leadership occurs seamlessly at all levels of the organization.

“So, leadership that champions this [SBP] and says this is important, which then enables faculty to develop the expertise to teach and create curricula and teach the content which then enables residency programs to make it a priority, to create space for the residents to do this learning. I think those are three are really what it’s about and you know the leadership obviously has to be…at the level of the association dean for GME or the Dean, but it also has to be the program director and hen it has to be the faculty and the chief residents have to champion this.” 

Participants spoke of the importance of both the environment they learned in and the larger culture of the system in which individuals practice. When speaking of the clinical learning environment (CLE), participants emphasized the importance of how that particular environment has the potential to deeply shape the learner’s journey to becoming a physician.

“I think the clinical environment, the clinical learning environment becomes really important right, you know if we start thinking about the impact of the hidden curriculum or the clinical learning environment, I know that's why the ACGME really put a lot of emphasis on safety and quality and other aspects of the learning environment. I think there's clear evidence that the learning environment can imprint on people and their practices are affected years down the road in terms of their own personal safety and in quality outcomes, and so I think the clinical environment where the learners are situated can be a real important facilitator of learning.” 

Put simply, another participant stated: “You know the saying goes, culture trumps everything else, and so you know the institutional learning environment is critical…it [emphasis on SBP] has to come from the top, there has to be a key message.”

Finally, participants described the formal recognition of SBP by the ACGME as a core competency as an essential precursor to organizational commitment to SBP. Despite formal recognition by the ACGME, organizations, such as hospital systems, have been slower to meaningfully adopt and implement SBP. Despite seeing SBP actualized at the organizational level, many interviewees described comprehensive organizational commitment as essential to this process.

“Most importantly, you know at the organizational level, you got to have your quality stuff in order… I think teach in a quality environment. And from the board of directors to the C-suite and the D-suite , you've got to have commitment to it. Culture that is really open and nurturing, and not blaming and punishing.” 

## Discussion

This qualitative study of individuals who helped found and refine the SBP competency over the past 20 years suggests that there is a need for co-production and learning health systems to advance SBP. As highlighted by the participants, SBP is, by its very nature, a competency highly dependent on the workplace environment. Therefore, SBP is a socially constructed competency in the CLE. Physicians cannot be expected to be competent in domains such as quality improvement, population health management, teamwork, or health information technology unless they work in a workplace that allows for, encourages, and facilitates practice and engagement in those competencies. SBP learning requires the presence of certain social workplace factors for learning to occur [22,23]. These environmental determinants of learning SBP include organizational culture, leadership support, the integration of best practices into clinical care, as in a learning health system, and co-production processes for healthcare delivery. With this understanding, we join other calls to urgently build interventions that impact the practice of clinical care in the CLE [24].

This study suggests the need for cooperation between health system and medical education leaders if SBP is to be fully realized for learners in the clinical learning environment, as additionally evidenced by several recent calls for the elimination of silos between healthcare systems and medical education [25-27]. There have also been descriptions of “bridging leader” roles that are necessary to advance some subsets of SBP, such as the quality and patient safety movement [27]. The results of this study suggest that there are far more opportunities for breaking down isolation and building co-production partnerships than in the quality improvement arena alone. As identified by the participants, co-production is about “users” and “providers” of a service coming together to jointly build a process to generate outcomes of shared interest and mutual benefit [20]. The “users” of healthcare education can be defined as individual patients, populations of patients, learners, or even the health system itself, since it uses education to develop a workforce. Therefore, if the goal is to advance SBP, resident education, and patient outcomes, we must consider bringing together all these participants in both the design of medical education curricula and clinical delivery systems.

A noteworthy finding was the emphasis international educators placed on the co-production that should occur between educators and community and/or government leaders. Indeed, our international educators stressed in this study, and in previous literature, the need for medical schools to demonstrate social accountability [28]. It is no longer acceptable to build education systems without first asking, “What do our communities need us to produce for them?” This question emphasizes the need for community leaders to be involved in our education structures and processes. This gives rise to a new set of “bridging” community roles that are outside of simply the partnership between educational and clinical institutions (Figure 1).

The participants collectively identified the need for co-production specifically between communities and health systems, communities and educational institutions, and health systems and educational institutions. This is summarized in Figure 1. However, this raises the question of exactly how such co-production might occur, which the participants did not specifically describe. The problem lies not only in differential expertise but also in fundamental differences in both focus and understanding that need to be bridged for co-production to truly occur. For example, while health system leaders might prioritize efficiency and revenue optimization, educational leaders might prioritize exposure and reflection, a process that could be at odds with efficiency. The way care occurs in clinical environments must be structured in a way that incorporates both business- and education-model worldviews. In the cultural competency movement of the late 1990s, the idea of “cultural brokers” was advanced to help doctors navigate the rocky shoals of medical relationships when their own medical culture was worlds apart from the culture of some patients. It may be that the bridging leader roles proposed by some of the participants in this study need to serve a function similar to that of cultural brokers, not only translating terms but also fostering understanding between the spheres that need to come together in a co-production model.

The act of increasing our joint work efforts and eliminating the barriers between educational and clinical systems is not enough. The participants were keen to share the critical role data transparency plays in transforming clinical healthcare systems into learning health systems. While co-production between partners is necessary, we must also be able to assess the outcomes of those partnerships and make changes quickly to address challenging areas. This requires healthcare and medical education systems to increase their data gathering, analysis, and sharing with one another and increase the use of standardized metrics to determine meaningful outcomes of the partnerships that may exist. This type of nimble, dynamic system is one in which SBP could truly flourish.

Lastly, we found that participants discussed faculty SBP knowledge as a strong facilitator of SBP implementation. Faculty who were “shining stars” or “early adopters” in their understanding and teaching of SBP were mentioned in most interviews. Therefore, systematically, a critical early task for GME leadership is to identify, support, and embrace these faculty so they may disseminate their SBP knowledge and skills not only to residents but also across the system. It has been documented that celebrating faculty who do not exhibit SBP knowledge and skills may lead to detrimental clinical imprinting on residents [28].

There are limitations to this study. We sought to include a diverse list of SBP experts; however, it could be that not all key individuals were included. To rectify potential gaps in our sampling strategy, we engaged in snowball sampling, asking participants to identify any additional names of potential interviewees in the area of SBP. This study only includes the perspectives of SBP experts and does not include the perspectives of “frontline” learners or program directors. We are currently engaging in additional studies to describe learners’ and program directors’ SBP views. Finally, there is potential for bias in this study due to the substantial background of the research team in systems-based practice. We attempted to balance this by including one member without significant training in systems-based practice; however, the interpretation of themes may still have been influenced by bias.

## Conclusions

The successful operationalization of SBP has proven difficult over the last 20 years. The complexity of this task also speaks to the presence of significant opportunity: the opportunity to improve the efficacy of SBP education for our learners and, thereby, the health care delivered to their patients. A fostering organizational culture, supportive leadership, co-production, and the establishment of learning health systems are all methods by which SBP implementation could potentially be improved.

## Appendices

Appendix 1

*Participant Interview Guide* 

Opening script read prior to each interview: Thank you for agreeing to participate in this research related to Systems-Based Practice. We have included the Summary Explanation of Research in the email communications with you. You have been invited to participate in this research study, and your participation is voluntary. Our research team includes physicians, educators, and researchers from Penn State College of Medicine, Geisinger Health, Allegheny Health Network, and Kaiser Permanente School of Medicine. The lead institution is the Penn State College of Medicine, and the primary data collection and analysis will occur under the leadership of Penn State investigators. The research study is exploring the concept of “Systems-Based Practice” in medical education. The questions you will be asked relate to your views on Systems-Based Practice and medical education. The data will be analyzed to identify themes and results to inform local and national initiatives. This interview is being recorded, and it will be transcribed after the interview. We are seeking to list all participants and their current/former roles in medical education in any scholarly work that results from the data, mainly to demonstrate credibility for the study. Although names will be listed, no opinion/statement will be directly tied to a specific name, i.e., any results or quotations will not be identifiable to a specific individual. There are no stipends for your participation in this work. There are no financial conflicts of interest on behalf of the researchers to disclose at this time. Do you have any questions? Do you consent to participate in this study?

Category 1: History/Evolution of SBP (“Backstory” of SBP)

Opening question: Can you describe your perspective on the origin of SBP?

What were the key issues that influenced the origin of SBP?

Probe: How did the SBP term come into being?

Probe: How did SBP come to be one of the six core ACGME competencies?

Can you tell us your perspective or share a story about the origin of SBP?

You have been identified as a person who has contributed to the founding or evolution of SBP. What were your motivations for going into the SBP realm?

How has SBP evolved over the past 20 years?

Probe: Can you describe any critical milestones or phases in the evolution of SBP over the past 20 years?

What is the ideal relationship between SBP and the other five ACGME core competencies?

At the start of SBP in the late 1990s, what were the design teams hoping SBP would lead to?

Probe, if not a “founder”: Can you speculate on the original intent and what the design teams were hoping SBP would lead to?

Probe: When you first got into SBP, what were you hoping SBP would be?

Probe: How is the reality different from what you were expecting?

Category 2: Teaching and Learning SBP: Implementation and Launch

Opening question: Can you describe the barriers and facilitators of SBP over the years and currently?

Can you talk about the barriers to educating about SBP?

Can you talk about the facilitators or enablers of educating about SBP?

Category 3: System Citizen: Operationalization of SBP and the Ultimate Goal of SBP

Opening question: What does the ideal resident physician do in the clinical workplace that would reflect that they are really great at SBP?

a. Tell me stories about your experiences with SBP and residents who are really good at SBP.

b. What behaviors would the physician demonstrate that would be the ideal outcome for SBP?

Category 4: Relationship to the Clinical Learning Environment: The Environment of SBP

Opening question: Can you please describe the relationship, if any, between the clinical learning environment, inclusive of sociocultural aspects, organizational components, and physician space, and the teaching and practice of SBP?

How have changes in clinical learning environments over the past 20 years influenced the learning and practice of SBP?

If the promise of SBP is to be fully realized, what, if anything, needs to change in current clinical learning environments?

What would be the ideal intersection between formal GME curricula and clinical learning environments?

Category 5: Future: Charting a Course

Opening question: Letting go of all practical considerations for the moment, what is your dream for SBP moving forward?

What advice do you have for workgroups that are seeking to evolve SBP?

How has the COVID-19 pandemic informed or changed the trajectory of SBP education?

What lies ahead for SBP? What needs to be done in the coming years to fully realize the potential of SBP?

What is missing? What haven’t we asked you that we should have asked you about SBP?
